# Supplementary material for: Prevalence of carbapenem resistance in Acinetobacter baumannii and Pseudomonas aeruginosa in sub-Saharan Africa: A systematic review and meta-analysis
Source: PLoS One. 2023 Nov 28;18(11):e0287762. doi: 10.1371/journal.pone.0287762 (PMC10684001; doi:10.1371/journal.pone.0287762)
Supplement: S1 Fig — (PDF) [file pone.0287762.s001.pdf]

```
                     proportion           95%-CI %W(random)
Ali et al. 2021          0.4722 [0.3041; 0.6451]        7.1
Kock et al. 2013         0.6289 [0.5248; 0.7248]        7.2
Kateete et al. 2016      0.0104 [0.0047; 0.0196]        7.3
Lowings et al. 2015      0.8511 [0.7628; 0.9161]        7.2
Nogbou et al. 2019       0.9500 [0.8872; 0.9836]        7.2
Kempf et al. 2012        0.0084 [0.0031; 0.0181]        7.3
Mohamed et al. 2019      0.0027 [0.0001; 0.0151]        7.3
Choonara et al. 2022     0.0058 [0.0016; 0.0147]        7.3
Musila et al. 2021       0.5625 [0.4118; 0.7052]        7.1
Olaitan et al. 2013      0.6000 [0.1466; 0.9473]        6.1
Kateete et al. 2017      0.0014 [0.0000; 0.0075]        7.3
Lakoh et al. 2020        0.0122 [0.0015; 0.0434]        7.2
Thomas et al. 2018       0.0209 [0.0105; 0.0370]        7.3
Abdeta et al. 2021       0.0037 [0.0012; 0.0087]        7.3

Number of studies combined: k = 14
Number of observations: o = 5791
Number of events: e = 322

                     proportion           95%-CI
Random effects model     0.1953 [0.0360; 0.4305]

Quantifying heterogeneity:
 tau^2 = 0.2263 [0.1169; 0.5913]; tau = 0.4757 [0.3418; 0.7690]
 I^2 = 99.1% [98.9%; 99.3%]; H = 10.59 [9.64; 11.64]

Test of heterogeneity:
       Q d.f.  p-value
 1458.44   13 < 0.0001

Details on meta-analytical method:
- Inverse variance method
- Restricted maximum-likelihood estimator for tau^2
- Q-Profile method for confidence interval of tau^2 and tau
- Freeman-Tukey double arcsine transformation
- Clopper-Pearson confidence interval for individual studies
```

S1 Fig. Analysis summary for CRAB
