# Supplementary material for: Prevalence of carbapenem resistance in Acinetobacter baumannii and Pseudomonas aeruginosa in sub-Saharan Africa: A systematic review and meta-analysis
Source: PLoS One. 2023 Nov 28;18(11):e0287762. doi: 10.1371/journal.pone.0287762 (PMC10684001; doi:10.1371/journal.pone.0287762)
Supplement: S2 Fig — (PDF) [file pone.0287762.s002.pdf]

```
                         proportion           95%-CI %W(random)
Choonara et al.(2021)        0.0259 [0.0154; 0.0407]        9.2
Abdeta et al.(2021)          0.0030 [0.0008; 0.0076]        9.3
Olowo-okere et al.(2019)     0.1182 [0.0645; 0.1936]        8.9
Mohamed et al.(2019)         0.0381 [0.0210; 0.0632]        9.2
Codjoe et al.(2019)          0.4595 [0.3645; 0.5567]        8.9
Musila et al.(2021)          0.2917 [0.1695; 0.4406]        8.4
Olaniran et al.(2021)        0.1651 [0.1313; 0.2037]        9.2
Kateete et al.(2017)         0.0041 [0.0008; 0.0119]        9.2
Adam and Elhag(2018)         0.1650 [0.1164; 0.2238]        9.1
Tadesse et al.(2019)         0.0115 [0.0055; 0.0211]        9.3
Kateete et al.(2016)         0.0115 [0.0055; 0.0211]        9.3

Number of studies combined: k = 11
Number of observations: o = 5771
Number of events: e = 241

                     proportion           95%-CI
Random effects model     0.0792 [0.0219; 0.1660]

Quantifying heterogeneity:
 tau^2 = 0.0494 [0.0235; 0.1577]; tau = 0.2222 [0.1533; 0.3971]
 I^2 = 97.9% [97.2%; 98.4%]; H = 6.88 [5.96; 7.94]

Test of heterogeneity:
      Q d.f.  p-value
 473.51   10 < 0.0001

Details on meta-analytical method:
- Inverse variance method
- Restricted maximum-likelihood estimator for tau^2
- Q-Profile method for confidence interval of tau^2 and tau
- Freeman-Tukey double arcsine transformation
- Clopper-Pearson confidence interval for individual studies
```

S2 Fig. Analysis summary for CRPA
