# Supplementary material for: Prevalence of carbapenem resistance in Acinetobacter baumannii and Pseudomonas aeruginosa in sub-Saharan Africa: A systematic review and meta-analysis
Source: PLoS One. 2023 Nov 28;18(11):e0287762. doi: 10.1371/journal.pone.0287762 (PMC10684001; doi:10.1371/journal.pone.0287762)
Supplement: Attachment — Submitted filename: RESPONSE TO REVIEWERS.docx [file pone.0287762.s004.docx]

January 25, 2023

Adriano Gianmaria Duse, MD

Academic Editor

PLOS ONE

Dear Professor Adriano Gianmaria Duse,

Thank you for your reply regarding our manuscript PONE-D-22-27050 titled ‘Prevalence of carbapenem resistance in Acinetobacter baumannii and Pseudomonas aeruginosa in sub-Saharan Africa: a systematic review and meta-analysis.’

We are grateful for you and the reviewers’ comments, and the positive evaluation of our work. We have revised and modified the manuscript to meet PLOS ONE’s style requirements, including those for file naming. We have reconstructed the minor overlapping text with the publication stated in the PLOS ONE decision letter and cited all sources used in this article.

Sincerely,

Margaret Toluwalayo Arowolo

Department of Microbiology

Faculty of Science

University of Lagos

Akoka-Yaba, Lagos

Nigeria

Journal Requirements: All the requirements stated have been revised and have been added to the manuscript.

When submitting your revision, we need you to address these additional requirements.

1. Please ensure that your manuscript meets PLOS ONE's style requirements, including those for file naming. The PLOS ONE style templates can be found at

<https://journals.plos.org/plosone/s/file?id=wjVg/PLOSOne_formatting_sample_main_body.pdf> and

<https://journals.plos.org/plosone/s/file?id=ba62/PLOSOne_formatting_sample_title_authors_affiliations.pdf>

2. We noticed you have some minor occurrence of overlapping text with the following previous publication(s), which needs to be addressed:

- <https://www.tandfonline.com/doi/full/10.1080/22221751.2019.1698273>

-<https://www.thefreelibrary.com/Prevalence+and+molecular+analysis+of+multidrug-resistant...-a0618470261>

In your revision ensure you cite all your sources (including your own works), and quote or rephrase any duplicated text outside the methods section. Further consideration is dependent on these concerns being addressed

3. In your Data Availability statement, you have not specified where the minimal data set underlying the results described in your manuscript can be found. PLOS defines a study's minimal data set as the underlying data used to reach the conclusions drawn in the manuscript and any additional data required to replicate the reported study findings in their entirety. All PLOS journals require that the minimal data set be made fully available. For more information about our data policy, please see <http://journals.plos.org/plosone/s/data-availability>.

Upon re-submitting your revised manuscript, please upload your study’s minimal underlying data set as either Supporting Information files or to a stable, public repository and include the relevant URLs, DOIs, or accession numbers within your revised cover letter. For a list of acceptable repositories, please see <http://journals.plos.org/plosone/s/data-availability#loc-recommended-repositories>. Any potentially identifying patient information must be fully anonymized.

Important: If there are ethical or legal restrictions to sharing your data publicly, please explain these restrictions in detail. Please see our guidelines for more information on what we consider unacceptable restrictions to publicly sharing data: <http://journals.plos.org/plosone/s/data-availability#loc-unacceptable-data-access-restrictions>. Note that it is not acceptable for the authors to be the sole named individuals responsible for ensuring data access.

We will update your Data Availability statement to reflect the information you provide in your cover letter.

4. Please ensure that you refer to Figures 5, 6 and 7 in your text as, if accepted, production will need this reference to link the reader to the figure.

5. We note that Figure 2 in your submission contain map images which may be copyrighted. All PLOS content is published under the Creative Commons Attribution License (CC BY 4.0), which means that the manuscript, images, and Supporting Information files will be freely available online, and any third party is permitted to access, download, copy, distribute, and use these materials in any way, even commercially, with proper attribution. For these reasons, we cannot publish previously copyrighted maps or satellite images created using proprietary data, such as Google software (Google Maps, Street View, and Earth). For more information, see our copyright guidelines: <http://journals.plos.org/plosone/s/licenses-and-copyright>. The map was not copyrighted but was created in R using the rnaturalearth package

We require you to either (1) present written permission from the copyright holder to publish these figures specifically under the CC BY 4.0 license, or (2) remove the figures from your submission:

 a. You may seek permission from the original copyright holder of Figure 2 to publish the content specifically under the CC BY 4.0 license.

We recommend that you contact the original copyright holder with the Content Permission Form (<http://journals.plos.org/plosone/s/file?id=7c09/content-permission-form.pdf>) and the following text:

“I request permission for the open-access journal PLOS ONE to publish XXX under the Creative Commons Attribution License (CCAL) CC BY 4.0 (<http://creativecommons.org/licenses/by/4.0/>). Please be aware that this license allows unrestricted use and distribution, even commercially, by third parties. Please reply and provide explicit written permission to publish XXX under a CC BY license and complete the attached form.”

Please upload the completed Content Permission Form or other proof of granted permissions as an "Other" file with your submission.

In the figure caption of the copyrighted figure, please include the following text: “Reprinted from [ref] under a CC BY license, with permission from [name of publisher], original copyright [original copyright year].”

 b. If you are unable to obtain permission from the original copyright holder to publish these figures under the CC BY 4.0 license or if the copyright holder’s requirements are incompatible with the CC BY 4.0 license, please either i) remove the figure or ii) supply a replacement figure that complies with the CC BY 4.0 license. Please check copyright information on all replacement figures and update the figure caption with source information. If applicable, please specify in the figure caption text when a figure is similar but not identical to the original image and is therefore for illustrative purposes only.

The following resources for replacing copyrighted map figures may be helpful:

USGS National Map Viewer (public domain): <http://viewer.nationalmap.gov/viewer/>

The Gateway to Astronaut Photography of Earth (public domain): <http://eol.jsc.nasa.gov/sseop/clickmap/>

Maps at the CIA (public domain): <https://www.cia.gov/library/publications/the-world-factbook/index.html> and <https://www.cia.gov/library/publications/cia-maps-publications/index.html>

NASA Earth Observatory (public domain): <http://earthobservatory.nasa.gov/>

Landsat: <http://landsat.visibleearth.nasa.gov/>

USGS EROS (Earth Resources Observatory and Science (EROS) Center) (public domain): [http://eros.usgs.gov/#](http://eros.usgs.gov/)

Natural Earth (public domain): <http://www.naturalearthdata.com/>

6. We note you have included a table to which you do not refer in the text of your manuscript. Please ensure that you refer to Table 1 and 2 in your text; if accepted, production will need this reference to link the reader to the Table.

7. Please review your reference list to ensure that it is complete and correct. If you have cited papers that have been retracted, please include the rationale for doing so in the manuscript text, or remove these references and replace them with relevant current references. Any changes to the reference list should be mentioned in the rebuttal letter that accompanies your revised manuscript. If you need to cite a retracted article, indicate the article’s retracted status in the References list and also include a citation and full reference for the retraction notice.

**Detailed response: we have addressed your editorial comments and responded to the comments by the reviewers as follows:**

**Response to Editor only (not for reviewers)**

Reviewer #1: General comments:
1. Absence of page numbers and line numbers.- Page numbers and line numbers have been added to the revised manuscript.
2. Genus and species are not consistently italicised in the main text.- This has been corrected
3. Figures should not be included in the text according to journal recommendations.- This has been corrected
4. Spelling errors : ''Acinetobacter baumanii'' for Acinetobacter baumannii'', Discussion - ''tecnics'' for ''techniques'' table 1 and 2: ''disc'' instead of ''disk''.- This has been corrected
5. Word in full prior to using abbreviations: e.g. AST for antimicrobial susceptibility testing.- This has been effected


Abstract:
1. Extra comma after blaimp under results section.- The extra comma has been removed
2. Add the funnel plot analysis to the methods.- This has been added
3. This sentence is not mentioned/explained in the main text ''Carbapenem-resistant A. baumannii prevalence based on
sample source gave estimates of 24% (95% CI; 6 – 49; I2=99%; P<0.01). ''- This has been explained.


Introduction:- These points have been observed and corrected in the revised manuscript
1. It would be beneficial to mention the global statistics with regards to the prevalence of carbapenem resistant
Acinetobacter baumannii and Pseudomonas aeruginosa here.
2. Prevalence of the common carbapenemase genes worldwide amongst CRAB and CRPA should be highlighted here.
3. Please reword this sentence: ''This has made infection therapy more difficult and expensive, especially against the
notorious Gram-negative bacteria''
4. Define ''ESKAPE group.''

Methods: These points have been observed and corrected in the revised manuscript
1. Need to define the study period accurately and include the months in 2012 and 2022 as the start and end dates.
2. Were all studies that meet the eligibility criteria, irrespective of language, included?


Results: These points have been observed and corrected in the revised manuscript

1. For the carbapenem resistant Pseudomonas aeruginosa (CRPA), a total of 11 articles reviewed, however, only 9 articles
mentioned (1st paragraph).
2. Figure 2, spelling error, ''Ghan'' for ''Ghana''. Only 10 articles instead of 11 mentioned for the CRPA.
3. Please recheck the Table 1: Nogbou et al, study aim - grammatical error.
Olaitan et al, study aim - spelling error (carbapenemase-encoding genes)
Table 2: Abdeta et al, study aim - spelling error ( non-susceptible)
Codejoe et al, eligibility criteria for article selection includes only human isolates of
carbapenem resistant Acinetobacter baumannii (CRAB) and CRPA. This study, however,
reflects the bacterial isolates from the hospital environment. Please recheck for eligibility.
4. Please use a key for wording AST below tables 1 and 2.
5. Please realign the wording in tables 1 and 2 for similarity.
6. Table 1 and table 2, please check and recalculate the % prevalence for the CRAB and CRPA as many are incorrect,
e.g. table 1, CRAB prevalence for Musila et al is written as 56% despite all (27) isolates of Acinetobacter baumannii
being CRAB. – The % prevalence of CRAB and CRPA were calculated as a ratio of of the drug-resistant strains to the total sample size x 100.
7. Table 2, Pseudomonas aeruginosa prevalence is missing in a few studies.
8. Figure 3 is not clear, very hazy.
9. The interpretation of the funnel plots are not described.
10. Please rewrite this sentence as it is ambiguous: ''Of the 14 articles analysed for the CRAB, (28.6%) were from South
Africa, Sudan and Uganda had 2(14.3%) each, while Ethiopia, Senegal Malawi, Kenya, Nigeria, and Sierra Leone had
one each''.


Discussion: These points have been observed and corrected in the revised manuscript

1. Please combine these sentences: ''Carbapenems are important broad-spectrum antimicrobials of last-resort, hence,
resistance to them signifies increased infection mortality, hospital stay duration, and cost of treatment (Friedman et al.,
2016). More importantly, when the infection is associated with notorious clinical pathogens such as A. baumanii and P.
aeruginosa.''
2.These sentences are redundant, please remove ''The true prevalence of these pathogens in sub-Saharan Africa is not well
known, especially the carbapenem-resistant strains. This study was carried out to determine the prevalence of the
carbapenem-resistant strains of A. baumanii and P. aeruginosa.''
3.This sentence does not make sense, please reword ''Thus, we believe that the current prevalence of antibiotic resistance
in A.baumannii infection is similar to the Sub-Saharan countries where these studies were carried out, most of which are
facing the same degree of severity of antibiotic resistance"'.
4. This sentence is confusing, advise rephrasing: A major strength of our study is that we included studies comprising of
non-disease-specific patients from patients, which ensured the representativeness of our estimates for these
institutions."


Conclusion: The sentence has been corrected and reworded in the revised manuscript

1. Sentence very lengthy, please rewrite/reword: ''Urgent epidemiological studies through comprehensive
surveillance of the pathogen at both hospital and community-based that will showcase the
prevalence of CRAB in the environment, animal/animal products, and the hospital is advocated
to be conducted with the inclusion of sub-Saharan African countries currently lacking this data.''


References: The references have been corrected to meet the journal requirements
1. References appear inconsistent. Was a reference manager used?
2. Advise vancouver style of intext referencing preferred by the journal.

Reviewer #2: Dear editor,
Thank you for inviting me to review this manuscript that analyses AMR for these two important organisms.
Comments and suggestions
Abstract
Background:
Based on the latest publications carbapenems resistance rate were high in A. baumannii and dominant in P. aeruginosa, and respectively they would not be considered as the drugs of choice in either organism. – “drug of choice” has been changed to “effective drugs against bacterial pathogens”
Introduction
In reference to Global burden of AMR, it would be advisable to mention attributable deaths to bacterial AMR. – mentioned in lines 42-44
Eligibility criteria, enhancement by recent publications.
There are some additional studies on ACIBA in the latest few months should be beneficial to mention, example: <https://journals.plos.org/plosone/article?id=10.1371/journal.pone.0271355>;
Results : article did not meet the eligibility criteria
Heading:
Prevalence of Carbapenem-resistant genes in A. baumanii and P. aeruginosa in sub-
Saharan Africa – The article discussed both the prevalence of CRAB and CRPA as well as genes. We think the current title suits that.
